# Y-chromosome variation in Basrah population

**DOI:** 10.25122/jml-2021-0281

**Published:** 2022-02

**Authors:** Bassim Muften Ohied, Adnan Issa Al-Badran

**Affiliations:** 1. Biology Department, College of Science, University of Basrah, Basrah, Iraq

**Keywords:** Basrah population, genetic diversity, Y chromosome, STR, forensic genetics

## Abstract

Y-chromosome DNA profiles are promising tools in population genetics and forensic science. Analysis of Y-chromosome variety was performed on a total of 191 unrelated males throughout different regions in Basrah. The Y-chromosome variety was explored utilizing 17 markers system. For the uniparental system, the large majority of the haplogroups observed in the Basrah population are (R1b, E1b1b, G2a, and J1) considered to have begun in the Middle East and to have later spread all over Western Eurasia. 30% of the Y-chromosomes, in all likelihood, represent landings from inaccessible distant geographic regions. The level of haplotype diversity and its implication for statistics are evaluated. The distinctive extent of long go genetic input observed for the Y chromosome shows that gene flow events to this area might have involved mainly males.

## Introduction

The human Y-chromosome is male explicit, and the connected Y-STRs situating on the non-recombining region of the Y chromosome has a patrilineal inheritance mode. It is only inherited from fathers to sons. They are transmitted unchanged except for the mutations, making the Y-STR haplotype very useful in paternal lineage testing [1, 2]. At present, Y-STRs are being widely utilized in forensic casework, especially in sexual assaults with high amounts of female DNA and paternity cases in the absence of an alleged father. They also play an important role in population genetics and human evolution studies. The main goal of the study was to understand the basic Y-chromosomal variation in Basrah and to evaluate the factors affecting the use of uniparentally inherited markers in Basrah for forensic casework.

## Material and Methods

Blood samples (191) were collected from unrelated, healthy male volunteers born and living in different parts of Basrah from different ages. DNA was extracted using the gSYNC™ DNA Extraction Kit Quick protocol by the Geneaid company.

### Y-STR genotyping

Fifteen single copy Y-STR loci (DYS19, DYS389I, DYS389II, DYS390, DYS391, DYS392, DYS393, DYS438, DYS439, DYS437, DYS448, DYS458, DYS456, DYS635, and Y-GATAH4) and a multicopy locus (DYS385a/b) were amplified with AmpFLSTR^®^_Yfiler™ PCR Amplification kit (Applied Biosystems, Foster City, CA, USA) according to the manufacturer’s instructions. The PCR products were genotyped with capillary array electrophoresis on ABI 3500 Genetic Analyzer (Applied Biosystems, Foster City, CA, USA). Genotyping was carried out using GeneMapperR _ ID-X (Applied Biosystems, Foster City, CA, USA). Allele designations were based on comparisons with the allelic ladder provided in the Yfiler kit.

### Statistical analyses

Y-STR haplotype data were set up for the analysis utilizing the MS Excel™ with Microsatellite Toolkit [3]. The essential parameters of molecular diversity were determined utilizing the Arlequin software ver. 3.1 [4]. Allele and haplotype frequencies were assessed by a simple gene counting method. The allele frequency of the multicopy locus DYS385 was examined as a blend of two alleles. Quality gene diversity (GD) of every locus and haplotype diversity (HD) was processed.

### Y-chromosome haplogroup prediction

The haplotypes of the 17 Y-STRs of 191 male individuals were submitted to Whit Athey’s Haplogroup Predictor (http://www.hprg.com/hapest5/index.html), with equivalent priors [5]. The most astounding probabilities were resolved as the derived haplogroups.

## Results

### Gene diversity

Gene diversity values for every Y-STR loci are given in Table 1. All-time low gene diversity (0.3547) was found in DYS392 locus, the best gene diversity (0.8461) was found in DYS385 locus, the lowest number of alleles (4) were observed in (DYS391, DYS439, DYS437, DYS438, loci respectively, while the highest number of alleles (9) was observed in (DYS385) locus.

**Table 1.** Observed number of alleles and gene diversity.

**Locus**	**Sample Size**	na*	ne*	h*	**I**
**DYS456**	191	6.0000	3.0788	0.6752	1.3221
**DYS 3891**	191	5.0000	1.9505	0.4873	0.8885
**DYS390**	191	7.0000	3.2054	0.6880	1.3982
**DYS38911**	191	7.0000	3.6354	0.7249	1.4963
**DYS458**	191	7.0000	4.5871	0.7820	1.6776
**DYS19**	191	8.0000	2.6168	0.6179	1.2462
**DYS385**	191	9.0000	6.4994	0.8461	1.9477
**DYS393**	191	6.0000	2.3135	0.5677	1.0564
**DYS391**	191	4.0000	1.9642	0.4909	0.8443
**DYS439**	191	4.0000	2.8077	0.6438	1.1692
**DYS635**	191	8.0000	3.4028	0.7061	1.5142
**DYS392**	191	7.0000	1.5497	0.3547	0.7958
**Y_GATA_H4**	191	5.0000	2.3914	0.5818	1.1228
**DYS437**	191	4.0000	2.1747	0.5402	0.9292
**DYS438**	191	4.0000	2.8173	0.6450	1.1738
**DYS448**	191	5.0000	2.5846::	0.6131	1.1916
**Mean**	191	6.0000	2.9737	0.6228	1.2359
**St. Dev**	191	1.6330	1.1988	0.1211	0.3160

na* – Observed number of alleles; ne* – Effective number of alleles; h* – gene diversity; I – Shannon's Information Index; The number of polymorphic loci is: 16; The percentage of polymorphic loci is: 100.00%.

Compared with the Turkish study on seventeen Y-STR loci from the Cukurova region of Turkey, the lowest gene diversity (0.5) was recorded in DYS 391, and the highest gene diversity (0.95) was found in DYS385. No vital variations were found with haplotype data of different Turkish populations [6].

For the Kuwaiti population, the lowest value of GD was observed for DYS437 (0.409), while the highest one (0.957) was found in DYS385. The lowest genetic diversity value for the Failaka Island population found in DYS392 (0.236), and the highest one (0.976) is presented by DYS385 [7].

Table 2 shows the mean number of pairwise differences (MPD) and the mean number of gene diversity for Basrah and neighboring populations. Iran has the highest mean number of gene diversity (0.69356) [8], Qatar shows the lowest (0.37274) [9]. Both Kuwait and Failaka Island shared a similar mean number of gene diversity (0.62578 and 0.64495, respectively) [7]. The mean number of pairwise differences for the Basrah population is calculated (16.745). The MPD values ranged from 5.521 for Qatar [9] to 10.911 for Iran [8]. The mean number of pairwise differences for Kuwait and Failaka Island are 8.951 and 8.775, respectively [7].

**Table 2.** The mean number of pairwise differences and gene diversity.

**Country and city**	**MPD**	**Gene diversity**	**References**
**Basrah**	16.745	0.6228	This study
**Kuwait**	8.951859 (±4.153213)	0.62578	[7]
**Failaka Island**	8.775362 (±4.195306)	0.64495	[7]
**Saudi Arabia**	5.276717(±2.563487)	0.47404	[10, 11]
**Iran**	10.911554(±4.974798)	0.69356	[8]
**Yemen**	5.942041 (±2.883168)	0.39659	[9]
**Qatar**	5.521739 (±2.702610)	0.37274	[9]
United Arab Emirates	7.121204 (±3.373326)	0.46178	[9]

### Allele frequency

The observed allele frequencies of the seventeen Y-STR loci are given in Table 3. The low frequency (0.0052) in allele (1) in DYS3891, DYS390 locus, allele (2) in DYS19, DSY393 allele (5) in DYS3891, allele (7) in DYS34, allele (8) in DYS635, allele (9) in DYS19 and DYS393. At the same time, high frequency was found in allele (3) in the DYS34 locus. The observed alleles in this study for all loci are between alleles (1) and (9). Compared with other populations like Turkish, the alleles observed were between (9) and (33) for all loci [6]. In this study, we did not observe intermediate or null alleles compared with the Turkish population, which found several intermediate alleles at DYS458 – 12.2, 16.2, 17.2, 18.2, 19.2, and 20.2, and one null allele was observed at DYS448 [6].

**Table 3.** Allele frequency.

**Locus Allele**	**DYS 456**	**DYS 3891**	**DYS 390**	**DYS 3891**	**DYS 458**	**DYS 19**	**DYS 385**	**DYS 393**	**DYS 33**	**DYS 439**	**DYS 635**	**DYS 34**	**Y_G_ATA**	**DYS 437**	**DYS 438**	**DYS 448**
**Allele 1**	0.0471	0.0052	0.0052	0.0105	0.0785		0.0105		0.0314	0.0942	0.0105	0.0052	0.0157	0.0052	0.2618	0.0681
**Allele 2**	0.2461	0.1257	0.0262		0.1361	0.0052	0.0157	0.0052	0.6492	0.4817	0.0890	0.0262	0.0681	0.6126	0.5026	0.2147
**Allele 3**	0.4764	0.6806	0.1152		0.3351	0.0209	0.0262	0.0314	0.2932	0.3246	0.4817	0.7958	0.5969	0.2670	0.1728	0.5654
**Allele 4**	0.1832	0.1832	0.4660	0.0157	0.2513	0.0785	0.1571	0.5916	0.0262	0.0995	0.1623	0.0209	0.2147	0.1152	0.0628	0.1257
**Allele 5**	0.0366	0.0052	0.0942	0.0942	0.0942	0.5497	0.1414	0.2670			0.1309	0.0785	0.1047			0.0262
**Allele 6**	0.0105		0.0262	0.2827	0.0942	0.2618	0.2042	0.0995			0.0995	0.0681				
**Allele 7**				0.4084	0.0105	0.0681	0.1571				0.0209	0.0052				
**Allele 8**				0.1204		0.0105	0.1309				0.0052					
**Allele 9**				0.0681		0.0052	0.1571	0.0052								

Table 4 shows the minimum and maximum allele frequency observed in this study. The low number of alleles was (4) at DYS33 with frequency (0.5091), DYS439 with frequency (0.3562), DYS437 with frequency (0.4598), DYS438 with frequency (0.3550), while the maximum number of alleles observed were (9) at DYS358 with frequency (0.1539). The minimum allele frequency of 0.1111 was observed at DYS385, while the maximum allele frequency of 0.9691 was observed at DYS33, DYS439, DYS437, DYS438.

**Table 4.** The minimum and maximum of allele frequency and the Statistics of Natural Selection.

**Locus**	**N**	**K**	**Obs. F**	**Min F**	**Max F**	Mean*	SE*	L95*	U95*
**DYS456**	191	6	0.3248	0.1667	0.9490	0.4622	0.0250	0.2413	0.8421
**DYS3891**	191	5	0.5127	0.2000	0.9590	0.5290	0.0298	0.2758	0.8990
**DYS390**	191	7	0.3120	0.1429	0.9391	0.4123	0.0212	0.2171	0.7630
**DYS38911**	191	7	0.2751	0.1429	0.9391	0.4162	0.0210	0.2191	0.7700
**DYS458**	191	7	0.2180	0.1429	0.9391	0.4113	0.0224	0.2192	0.7862
**DYS19**	191	8	0.3821	0.1250	0.9294	0.3710	0.0171	0.1976	0.7012
**DYS385**	191	9	0.1539	0.1111	0.9197	0.3417	0.0157	0.1863	0.6643
**DYS393**	191	6	0.4323	0.1667	0.9490	0.4660	0.0278	0.2428	0.8510
**DYS33**	191	4	0.5091	0.2500	0.9691	0.5989	0.0324	0.3207	0.9486
**DYS439**	191	4	0.3562	0.2500	0.9691	0.6025	0.0335	0.3251	0.9387
**DYS635**	191	8	0.2939	0.1250	0.9294	0.3725	0.0187	0.1951	0.7259
**DYS34**	191	7	0.6453	0.1429	0.9391	0.4110	0.0209	0.2144	0.7691
**Y_GATA_H4**	191	5	0.4182	0.2000	0.9590	0.5258	0.0299	0.2665	0.8990
**DYS437**	191	4	0.4598	0.2500	0.9691	0.6018	0.0326	0.3267	0.9387
**DYS438**	191	4	0.3550	0.2500	0.9691	0.5996	0.0318	0.3198	0.9385
**DYS448**	191	5	0.3869	0.2000	0.9590	0.5293	0.0308	0.2712	0.8990

These statistics were calculated using 1000 simulated samples.

### Chromosome STRs Haplotype

We compared the Haplotypes found in this study with seven different populations: Tunis (n=81) [10], German (n=88), Indian (n=25), Chinese (n=36), Italians (n=100) [11], Japanese (n=161) [12], and Turkish (n=245) [13] (Table 5).

**Table 5.** Comparison of the haplotypes in different human population groups.

**Population group**	**Basrah**	**Iraq**	**Tunis**	**German**	**Italy**	**China**	**India**	**Turkish**
**No. of individuals**	191	100	105	88	100	36	154	281
**No. of haplotypes**	161	96	81	77	82	34	125	245

### Y-chromosome haplogroup prediction

The results of Y haplogroup predictions and their probabilities for the Basrah population are shown in Table 6. The most common haplogroups in Basrah are R1b (20.5%), E1b1b (14.0%), G2a (11.0%) and J1 (10.8%), and 17% are (J2a1b (5%), J2a1h (2%), J2a1 x j2a1-bh (4%), J2b (6%). The most common haplogroups in Kuwait are J1 (37%), R1a (11%), and E1b1b (7%), while the most common haplogroups in Failaka are J1 (20%) and E1b1b (17%) [7]. Y haplogroups J2b, J2a1xJ2, G2a, were found in high frequencies in Failaka Island (13%) and Kuwait (1%), (3%), and (3%), respectively. The haplogroups H, T were observed in this study (10.5%), (0.7%), in Kuwait (3%), (4%), respectively, and not present in Failaka Island [7]. The most frequent haplogroups in the Caucasus were F*, G*, and J2 together. The frequency of these three haplogroups was 0.53–1.00. The frequency of haplogroup (I1) in this study was 1.7, (I2a (xI2a1), 6.5), (I2a1,2.0), (I2b (xI2b1), 0.4), (I2b1,1.6) compared with Darginians (0.58), Abkhazians (0.33), and North Ossetians from Ardon (0.32) [14]. The frequency of J2 in this study, J2a1b was 5.0%, J2a1h,2.0%, J2a1 x J2a1-bh,4.5%. J2b,6.5 compared with the Georgian population from Kazbegi frequency of haplogroup J2(0.72) [15]. The haplogroup G2a frequency observed in this study was 11.0%. Compared with other populations, the common Caucasus haplogroup, G, is rare or absent in Europe and Turkish and Lebanese groups [16], but not in populations from Tehran and Isfahan (frequency of 0.1 and 0.2 respectively. The most common haplogroups in Basrah are R1b (20.5%). HaplogroupR1, which is common in Western and Central Europe, is observed mostly in the South Caucasus [16].

**Table 6.** Haplogroup probability.

**Haplo- group**	**Probability %**
**E1b1a**	1.5
**E1b1b**	14.0
**G2a**	11.0
**G2c**	0.3
**H**	1.5
**I1**	1.7
**I2a (xI2a1)**	6.5
**I2a1**	2.0
**I2b (xI2b1)**	0.4
**I2b1**	1.6
**J1**	10.8
**J2a1b**	5.0
**J2a1h**	2.0
**J2a1 x J2a1-bh**	4.5
**J2b**	6.5
**L**	2.0
**N**	0.3
**Q**	3.2
**R1a**	4.0
**R1b**	20.5
**T**	0.7

## Conclusions

DYS385 had the highest diversity (GD=0.8461), while loci DYS392 had the lowest (GD=0.3547). The mean number of pairwise differences of the Basrah population is 16.745, and the gene diversity is. 0.6228. There was a low frequency (0.0052) in allele (1) in DYS3891, DYS390 locus, allele (2) in DYS19, DSY393 allele (5) in DYS3891, allele (7) in DYS34, allele (8) in DYS635, allele (9) in DYS19 and DYS393. High frequency was found in allele (3) in the DYS34 locus. High gene frequency 0.6453 was found in locus DYS34, and the low gene frequency was found in locus DYS385, 0.1539. The most common haplogroups in Basrah are R1b (20.5%), E1b1b (14.0%), G2a (11.0%) and J1 (10.8%).

## Acknowledgments

### Conflict of interest

The authors declare no conflict of interest.

### Ethical approval

This study was approved by the Scientific Committee of the Biology Department, College of Science, University of Basrah (approval number: 7/54/4591,7/8/2018).

### Consent to participate

Written informed consent was obtained from the participants before obtaining the sample.

### Data availability

Further data is available from the corresponding author on reasonable request.

### Personal thanks

I wish to express my deepest gratitude to the Head of Biology Department Dr. Munaff J.Abd Al-Abbas and to the Cell and Biotechnology researchers unit in the College of Science, Basrah University and Dr. Dhamia Kassim Suker, for their assistance.

### Authorship

AIA suggested the main aims and objectives of the research. BMO contributed to most experimental work and data analysis. Both authors, AIA and BMO wrote and reviewed the manuscript.
